# Effect of supersaturated dissolved oxygen on growth-, survival-, and immune-related gene expression of Pacific white shrimp (*Litopenaeus vannamei*)

**DOI:** 10.14202/vetworld.2024.50-58

**Published:** 2024-01-08

**Authors:** Songwut Patkaew, Sataporn Direkbusarakom, Ikuo Hirono, Suwit Wuthisuthimethavee, Sorawit Powtongsook, Chettupon Pooljun

**Affiliations:** 1 Center of Excellence for Aquaculture Technology and Innovation, School of Agricultural Technology and Food Industry, Walailak University, Nakhon Si Thammarat, Thailand; 2 Graduate School of Marine Science and Technology, Tokyo University of Marine Science and Technology, Konan 4-5-7, Minato-ku, Tokyo 108-8477, Japan; 3 National Center for Genetic Engineering and Biotechnology, National Science and Technology Development Agency, Pathum Thani, Thailand; 4 Department of Marine Science, Center of Excellence for Marine Biotechnology, Faculty of Science, Chulalongkorn University, Bangkok, Thailand; 5 Akkhraratchakumari Veterinary College, Walailak University, Nakhon Si Thammarat, Thailand; 6 Research Center on One Health, Walailak University, Nakhon Si Thammarat, Thailand

**Keywords:** gene expression, growth performance, molting, pacific white shrimp, supersaturated dissolved oxygen

## Abstract

**Background and Aim:**

Oxygen concentration is an essential water quality parameter for aquaculture systems. Recently, supersaturated dissolved oxygen (DO) has been widely used in aquaculture systems to prevent oxygen depletion; however, the long-term effects of supersaturated DO exposure on aquatic animals have not been studied. In this study, we examined the effects of supersaturated DO on the growth, survival, and gene expression of Pacific white shrimp (*Litopenaeus vannamei*).

**Materials and Methods:**

Specific pathogen-free shrimp with a body weight of 8.22 ± 0.03 g were randomly assigned to two groups with four replicates at a density of 15 shrimps per tank. Shrimp were cultivated in recirculating tanks containing 50 L of 15 ppt seawater in each replicate. Oxygen was supplied at 5 mg/L to the control tanks using an air microbubble generator and at 15 mg/L to the treatment tanks using a pure oxygen microbubble generator. Shrimp were fed commercial feed pellets containing 39% protein at 4% of their body weight per day for 30 days. Average daily growth (ADG) and feed conversion ratio (FCR) were determined on days 15 and 30. Shrimp molting was measured every day. Individual hemolymph samples were obtained and analyzed for total hemocyte count, differential hemocyte count, and expression of growth- and immune-related genes at the end of the experiment.

**Results:**

Long-term exposure to supersaturated DO significantly affected shrimp growth. After 30 days of supersaturated DO treatment, the final weight and ADG were 14.73 ± 0.16 g and 0.22 ± 0.04, respectively. Shrimp treated with normal aeration showed significantly lower weight (12.13 ± 0.13 g) and ADG (0.13 ± 0.00) compared with the control group. FCR was 1.55 ± 0.04 in the treatment group and 2.51 ± 0.09 in the control group. Notably, the shrimp molting count was 1.55-fold higher in the supersaturated DO treatment than in the supersaturated DO treatment. The expression of growth-related genes, such as alpha-amylase, cathepsin L, and chitotriosidase, was 1.40-, 1.48-, and 1.35-fold higher, respectively, after supersaturated DO treatment. Moreover, the treatment increased the expression of anti-lipopolysaccharide factor, crustin, penaeidin3, and heat shock protein 70 genes by 1.23-, 2.07-, 4.20-, and 679.04-fold, respectively, compared to the controls.

**Conclusion:**

Supersaturated DO increased growth and ADG production and decreased FCR. Furthermore, enhanced immune-related gene expression by supersaturated DO may improve shrimp health and reduce disease risk during cultivation.

## Introduction

Dissolved oxygen (DO) is a key indicator of water quality in aquaculture, particularly in intensive aquaculture systems. It is essential to maintain adequate levels of DO in pond water for the growth and survival of aquatic organisms. An oxygen concentration exceeding 5 mg/L is generally recommended for shrimp culture. DO directly affects aquaculture production in terms of survival and growth rate [1–4]. Prediction of DO levels and efficient aeration systems are required for adequate DO management [5–7]. A previous study found that oxygen concentrations below 2 mg/L reduced the growth of Pacific white shrimp [8]. In another study, white shrimp grown under more than 4 mg/L DO grew significantly faster than shrimp maintained under lower oxygen concentrations (2–4 mg/L) [9]. Similarly, a study reported that 5–7 mg/L oxygen from a nanobubble device resulted in faster shrimp growth than typical bubble aeration at 3–6 mg/L [10]; this was due to the low oxygen concentrations directly affecting the swimming ability and physiological responses of shrimp [11].

Oxygen is required for shrimp respiration and other metabolic processes. Various aeration systems, such as nanobubble on-site oxygen generation systems [12, 13] and pure oxygen contact systems with aeration control facilitated by DO sensors [14], have been devised to enhance the DO levels while conserving energy for shrimp cultivation. Solar-powered aerators have also been developed [15]. Therefore, highly efficient aeration for oxygen control has become essential for intensive and superintensive shrimp farming [2]. Mechanical aerators include floating long- and short-arm aerators, partially submerged paddlewheels, vertical turbines, diffusers, and Venturi aerators. These aerators are widely used in shrimp farming [2, 16]. Alternative techniques, such as microbubble and nanobubble generators, have been developed for a more efficient supply of DO in shrimp ponds. Small-sized bubble generators can increase DO levels up to 9.0–10.8 mg/L [17]. The application of nanobubble oxygen in an indoor raceway shrimp pond has been suggested to substantially increase shrimp growth as it led to doubled total harvest and productivity, whereas the total *Vibrio* count and feed conversion ratio (FCR) were decreased [10]. This phenomenon is probably due to an increase in shrimp feed consumption rate [17]. In addition to assessing shrimp growth and survival rates, the analysis of gene expression in shrimp cultures under environmental stress provides insights into growth mechanisms. Notably, genes encoding alpha-amylase [18], cathepsin L [19], and chitotriosidase 1 [20] play crucial roles in digestive function, intracellular protein turnover, and chitin metabolism, respectively, and influence assimilation, body weight, growth rate, and feed efficiency in shrimp. Low DO levels have a direct negative effect on the shrimp immune system and have been shown to depress phagocytosis and bacterial clearance efficiency in black tiger shrimp [21]. Low oxygen levels (<2 mg/L) suppress white shrimp immune activity by decreasing total hemocytes, phagocytosis, bactericidal activity, phenol oxidase activity, and superoxide dismutase activity [9]. In response to bacterial lipopolysaccharides (LPS), hemocytes rapidly degranulate and release LPS with pronounced antibacterial activity against Gram-negative bacteria [22]. Environmental stressors, such as supersaturated DO and ammonia, have also been observed in the degradation of antimicrobial peptides such as crustin and penaeidins [23, 24]. Peptides responsive to environmental stress can serve as stress indicators, and stress-related proteins, such as heat shock protein 70 (Hsp70), play crucial roles in repairing denatured proteins and stimulating the innate immune system under various conditions [25, 26].

Recently, supersaturated DO has been widely used in aquaculture systems to prevent oxygen depletion, especially in recirculating aquaculture systems (RAS), an advanced method that focuses on water recycling within a closed system. In RAS, water is continuously circulated through tanks or ponds, providing a controlled environment for the cultivation of aquatic organisms such as fish or shrimp. RAS frequently requires an increase in DO levels for several reasons, such as high stocking density, limited exchange with atmospheric oxygen, biological processes, and temperature. RAS operators frequently use aeration devices or other oxygenation techniques to supplement the DO levels in the water to address these factors and ensure the thriving of aquatic species; however, the long-term effects of supersaturated DO on aquatic animals, especially shrimp, have yet to be studied.

In this study, we investigated the effects of supersaturated DO on Pacific white shrimp (*Litopenaeus vannamei*) growth, survival rates, expression of growth-related, immune-related, and stress tolerance-related genes in a recirculating system. Our results can be used to increase the efficiency of shrimp farming by supersaturated DOs.

## Materials and Methods

### Ethical approval

All animal experiments were approved by the Animal Ethics Committee, Walailak University (protocol no. WU-AICUC-64031).

### Study period and location

The study was conducted from February 2022 to November 2022 at the Center of Excellence for Aquaculture Technology and Innovation, School of Agricultural Technology and Food Industry, Walailak University.

### Shrimp acclimatization

A total of 130 juvenile Pacific white shrimp, aged 45 days, with an average weight of 8.22 ± 0.03 g, were obtained from NSW Farm, Nakhon Si Thammarat, Thailand. The shrimp were allowed to acclimate for 7 days in a 4000-L cement tank with a RAS containing 2500 L aerated natural seawater (15 ppt salinity, pH 7.8, 28 ± 1°C) at the Center of Excellence for Aquaculture Technology and Innovation, Walailak University. During this acclimation period, ten shrimp were randomly sampled to screen for diseases, including acute hepatopancreatic necrosis disease, covert mortality nodavirus, infectious hypodermal and hematopoietic necrosis virus, Taura syndrome virus, white spot syndrome virus (WSSV), and yellow head virus, as described previously [27–32]. The remaining shrimp were fed a commercial diet (Nanami No. 2) with 39% crude protein (Thai Union Feedmill, Thailand) at 4% of body weight three times daily.

### Experimental design and shrimp treatment

After acclimatization, 120 shrimps were divided into two groups (n = 60, each) with four replicates each. Fifteen shrimp were placed in individual 0.3 × 0.5 × 0.4 m^3^ rectangular plastic tanks filled with 50 L of natural seawater (15 ppt salinity, pH 7.8, 28 ± 1°C) with a RAS and a trickling biofilter. Oxygen was supplied at 5 mg/L in the control tanks using an air microbubble generator (WU Oxygen Microbubble Generator 1) and at 15 mg/L in the treatment tanks using pure oxygen flow through a WU Oxygen Microbubble Generator 1. The microbubble diameter was approximately 92 ± 3.14 μm. All shrimps were fed the same diet at 4% of body weight three times daily for 30 days throughout the experimental period. The water was changed daily by 10% of the tank volume.

### Water quality analysis

Throughout the experiment, water quality parameters, including alkalinity, salinity, pH, DO, and temperature, were monitored daily. Calcium, magnesium, ammonia, nitrite, nitrate, and total suspended solids (TSS) were measured every 3 days. The analytical methods used to assess these parameters are listed in Table-1 [33, 34].

**Table-1:** Water quality analysis methods.

Water parameter	Instrument/method
DO	DO meter (YSI, model Pro 20i.)
Temperature	DO meter (YSI, model Pro 20i.)
pH	pH meter (Orion: 420A)
Alkalinity	American Public Health Association [33]
Ammonia	Strickland and Parsons [34]
Nitrite	Strickland and Parsons [34]
Nitrate	Strickland and Parsons [34]
Orthophosphate	Strickland and Parsons [34]
Magnesium	American Public Health Association [33]
Calcium	American Public Health Association [33]
Total suspended solids	American Public Health Association [33]

DO=Dissolved oxygen

### Growth performance and survival rate analysis

The effect of supersaturated DO on shrimp growth, survival, and molting rates was determined. Before feeding on days 0, 15, and 30, shrimp body length and weight were individually measured using a digital scale to record the average daily growth (ADG) and FCR. At the end of the experiment, we counted the shrimp to determine the survival rate. In shrimps, molting is an intrinsic physiological process that occurs naturally during growth. Exuviae (i.e., shed carapace or exoskeleton) were used to assess the frequency of shrimp molting. This was achieved through daily systematic counting of shed carapaces in each tank; the numbers were recorded every morning throughout the experiment.

### Bacterin preparation and injection

*Vibrio parahaemolyticus* was prepared as described previously by Pooljun *et al*. [35]. *V*. *parahaemolyticus* was prepared from the hepatopancreas of moribund shrimp, and infection was confirmed by polymerase chain reaction (PCR) using TUMSAT-Vp3 primers [27]. Bacteria were cultured in tryptic soy broth (Difco, Detroit, MI, USA) at 30°C overnight under shaking at 200 rpm to produce cell suspensions. *V. parahaemolyticus* cells were harvested during the log phase (OD600 = 1.0; 1 × 10^8^ cells/mL) by centrifugation at 7494× *g* and 4°C for 5 min. The cells were washed twice using an equal volume of normal saline and then killed by incubation in 0.3% formalin for 24 h at 25°C. Formalin-inactivated bacteria were washed thrice with normal saline. We confirmed the absence of culturable cells using tryptic soy agar (Difco) and the absence of colony formation after incubation at 30°C for 16–18 h. Formalin-inactivated bacterin was diluted to 10^6^ cells/mL with normal saline before administration to the shrimp.

Two shrimp were randomly selected from each tank (n = 16 in total) on day 30 of the experiment and intramuscularly injected with 0.1 mL of the formalin-deactivated bacterin on the ventral side of the sixth abdominal segment.

### Total hemocyte count (THC) and differential hemocyte count (DHC)

Shrimp were anesthetized on ice for 30 s after treatment with formalin-deactivated bacterin for 6 h before hemolymph collection. Then, 0.2 mL hemolymph per individual was collected from the third walking leg using a 24-gauge hypodermic needle on a 1-mL syringe that was filled with 0.2 mL of anticoagulant (115 mM glucose, 385 mM NaCl, and 27 mM trisodium citrate; pH 7.5) for the THC and DHC. For THC, 50 μL of anticoagulated hemolymph was fixed for 10 min in 150 μL of 4% formalin, and then, 20 μL of fixed hemolymph was placed in a Neubauer Hemocytometer to record the THC (i.e., the number of hemocytes per mL), as described previously by Sritunyalucksana *et al*. [36]. For DHC, Bauchau and Mengeot [37] methods were used to measure the percentage of granular hemocytes and hyaline cells. To this end, 50 μL of anticoagulated hemolymph was mixed with 10% formalin at a 1:1 ratio. One drop of the mixture was placed on a clean microscopic slide, smeared, air-dried, fixed in absolute methanol for 6 min, and stained with 10% Giemsa stain diluted with phosphate-buffered saline, pH 7.4 for 10 min. The slide was then washed thoroughly with running water and allowed to dry before counting. We counted 100 stained hemocytes in randomly selected fields with three differential slides per sample subjected to counting. This analysis was conducted at 1000-fold magnification using a light microscope (Nikon Eclipse E100 Biological Microscope; Nikon, Tokyo, Japan). According to Tsing *et al*. [38], differential hemocytes were then characterized according to the presence or absence of cytoplasmic granules as a simple criterion. DHCs were calculated using the following equation:


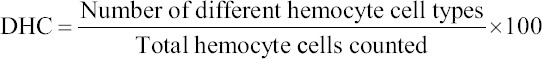


Each shrimp’s THC and DHC count was assess using triplicate counts.

### Gene expression analysis

We assessed the effect of supersaturated DO on specific gene expression in hemocytes, muscle, and hepatopancreas samples (Table-2).

**Table-2:** Sample material for total RNA extraction and target genes.

Tissue	Target gene (s)
Hemocytes	Immune-related genes: Anti-lipopolysaccharide factor, crustin, and penaeidin 3
Muscle of the second swimming leg	Growth-related genes: Alpha-amylase, cathepsin L, and chitotriosidase
Hepatopancreas	Stress tolerance-related gene: *Litopenaeus vannamei* heat shock protein 70

### Total RNA isolation

To assess immune-related gene expression, total RNA was extracted from hematocytes obtained through centrifugation of the residual hemolymph from THC and DHC experiments at 5974× *g* and 4°C for 10 min. We used the hepatopancreas and muscle tissue of these shrimps to analyze stress tolerance and growth-related genes, respectively. Total RNA was extracted using PureZOL™ RNA Isolation Reagent (Bio-Rad, Hercules, CA, USA) according to the manufacturer’s instructions.

A NanoDrop spectrophotometer (NanoDrop™ 2000/2000c Spectrophotometer; Thermo Fisher Scientific, Waltham, MA, USA) was used at wavelengths of 260 and 280 nm to determine the total RNA concentration in each sample. Total RNA of each sample (260 nm/280 nm ratio ranging from 1.8 to 2.0) was used for cDNA synthesis.

### cDNA synthesis and gene expression analysis

One microgram of total RNA was used to synthesize cDNA using an iScript™ cDNA synthesis kit (Bio-Rad) according to the manufacturer’s instructions, and cDNA products were stored at −80°C for gene expression analysis by quantitative real-time-PCR (RT-PCR). The nucleotide sequence of each gene was obtained according to the gene designation from the National Center for Biotechnology Information database. Primer sequences for each gene were designed using Primer 7 Plus software (http://tinyurl.com/4zu37tej; Table-3).

**Table-3:** Primers used for the real-time PCR assay of seven genes and one internal control (β-actin gene) of Pacific white shrimp (*Litopenaeus vannamei*).

Target gene	GenBank accession number	Forward/reverse primer sequence	Amplicon size (bp)
Housekeeping gene			
β-actin	AF300705.2	5’- CCACGAGACCACCTACAAC-3’ 5’- AGCGAGGGCAGTGATTTC-3’	142
Immune-related genes			
Anti-lipopolysaccharide factor	MF135540.1	5’- CATTCGGCCTTGACTTCG-3’ 5’- ATCCAGGACACCACATCCTG-3’	260
Crustin	AY486426.1	5’- GAGGGTCAAGCCTACTGCTG-3’ 5’- ACTTATCGAGGCCAGCACAC-3’	157
Penaeidin 3	DQ206403.1	5’- CACCCTTCGTGAGACCTTTG-3’ 5’- AATATCCCTTTCCCACGTGAC-3’	141
Growth-related genes			
Alpha-amylase	XM_027369804.1	5’- GCCATATCCAGCGGAGAGTA-3’ 5’- CGCCGAAGTTGTTGAGGTA-3’	150
Cathepsin L	Y13924.1	5’- GTCGACTGCTCCGACAAGTT-3’ 5’- AAGAGTCCTCGGTGTCGATG-3’	438
Chitotriosidase 1	XM_027371275	5’- CCAGACACTCGGAGTGATA-3’ 5’- TCTCCTACACATGCACAACC-3’	200
Stress tolerance-related genes			
Heat shock protein 70	JQ736788.1	5’- CTCCTGCGTGGGTGTGTT-3’ 5’- GCGGCGTCACCAATCAGA-3’	120

PCR=Polymerase chain reaction

A CFX connect RT-PCR detection system (Bio-Rad) was used to measure the expression of target genes. A total of 20 μL containing 2 μL diluted cDNA, 4 μL 5 × HOTFIREPol EvaGreen quantitative PCR Mix Plus (ROX) (Solis Biodyne, Tartu, Estonia), 0.5 μL of forward and reverse primers, and 13 μL of PCR-grade water was used for amplification.

Thermocycling was conducted as follows: initial denaturation at 95°C for 15 min, followed by 40 cycles of 95°C for 20 s, 60°C for 20 s, and 72°C for 30 s. Dissociation analysis was performed to determine the melting temperature of a single amplification product at the end of each PCR cycle. Each sample was examined in triplicate, and the relative expression was determined using the comparative threshold cycle method (2^-ΔΔCT^ method) [39], with the β-actin gene as a reference for normalization. The data are presented as relative mRNA levels (mean ± standard error).

### Statistical analysis

Statistical package for the social sciences version 22.0 (IBM, Armonk, NY, USA) was used for all statistical analyses. An independent sample t-test was conducted to test differences between treatment and control groups in growth performance, survival rate, and immune parameters, including THC, DHC, and immune-related gene expression. p < 0.05 is considered statistically significant. A one-way analysis of variance at a significance level of 5% was used to evaluate differences in growth performance, survival rate, and immune parameters, including THC, DHC, and immune-related gene expression, between the treatment and control groups using a mean comparison test.

## Results

### Water quality analysis

Most of the physical and chemical water quality parameters in the control and oxygen-saturated groups were within the optimal ranges for *L. vannamei* cultivation (Table-4), and no significant differences were observed in the water quality parameters between the control and treatment groups. The minimum and maximum DO concentrations were in the range 4.90–5.41 and 15.12–15.89 mg/L, respectively, throughout the experiment.

**Table-4:** Water quality parameters during the experiment.

Water quality	Control (DO 5 mg/L)	Treatment (DO 15 mg/L)
pH	8.23 ± 0.01	8.22 ± 0.01
Temperature (°C)	28.16 ± 0.09	27.88 ± 0.10
Alkalinity (mg/L as CaCO_3_)	179.19 ± 6.41	178.50 ± 5.58
Calcium (mg/L)	202.25 ± 3.59	201.46 ± 2.87
Magnesium (mg/L)	591.8 ± 24.45	590.3 ± 24.05
Total ammonia (mg NH_3_-H/L)	0.83 ± 0.81	0.76 ± 0.72
Nitrite (mg NO_2_-N/L)	0.12 ± 0.09	0.10 ± 0.08
Nitrate (mg NO_3_-N/L)	5.32 ± 4.16	5.33 ± 4.14
Orthophosphate (mg/L)	0.22 ± 0.06	0.22 ± 0.05
TSS (mg/L)	11.30 ± 1.89	11.09 ± 1.70

Values are mean ± standard deviation of four replicates per group. TSS=Total suspended solids, DO=Dissolved oxygen

### Shrimp growth

All shrimps in the control and treatment groups survived throughout the 30-day experimental period (Table-5). Table-5 shows the weight and length of the shrimp. Weight gain in the first 15 days did not differ significantly between the groups (p > 0.05); however, at the end of the experiment, shrimp exposed to supersaturated DO showed 1.21-fold higher weight than the aerated control. ADG was significantly higher in the treatment group than in the control group (p < 0.05). The body length in the treatment group (12.13 ± 0.13 cm) was significantly larger (p < 0.05) than that of the control group (14.73 ± 0.16 cm), which was distinguishable by eye from day 15.

**Table-5:** Effect of supersaturated DO on shrimp weight, length, survival rate, ADG, total feed intake, and FCR.

Day	Average shrimp weight (g)		Average shrimp length (cm)	
	
	Control (DO 5 mg/L)	Treatment (DO 15 mg/L)	Control (DO 5 mg/L)	Treatment (DO 15 mg/L)
0	8.22 ± 0.03^a^	8.22 ± 0.02^a^	10.00 ± 0.14^a^	9.94 ± 0.08^a^
15	10.29 ± 0.57^a^	11.11 ± 0.45^a^	10.46 ± 0.19^a^	10.94 ± 0.09^b^
30	12.13 ± 0.13^a^	14.73 ± 0.16^b^	10.96 ± 0.19^a^	11.54 ± 0.22^b^
Survival rate (%)	100^a^	100^a^		
*ADG	0.13 ± 0.00^a^	0.22 ± 0.04^b^		
*Total feed intake (g)	147.13 ± 2.09^a^	151.75 ± 1.04^a^		
*FCR	2.51 ± 0.09^a^	1.55 ± 0.04^b^		

^a,b^Different superscript letters in rows indicate significant differences (t-test).

* At the end of the experiment, the ADG, total feed intake, and feed conversion ratio were calculated to evaluate the growth performance of the shrimp in the treatment and control groups. DO=Dissolved oxygen, FCR=Feed conversion ratio, ADG=Average daily growth

**Figure-1:**
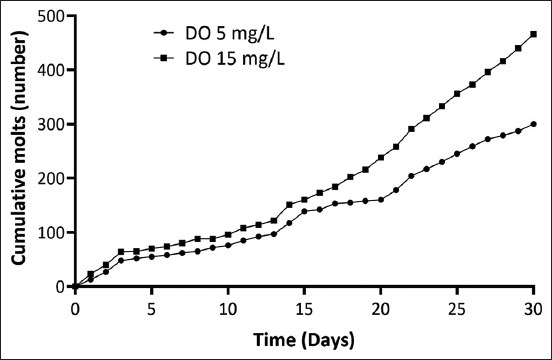
Effect of supersaturated dissolved oxygen (DO) on the molting rates of Pacific white shrimp (*Litopenaeus vannamei*) in the experimental and control groups over the 30-day experimental period.

Shrimp molting was regularly monitored during the experiment. Shrimp in supersaturated DO had a significantly higher molting rate (1.55-fold; p < 0.05) than those in the control group. As shown in Figure-1, the accumulation of shrimp crusts after 15 days was higher than that in the initial period. The increase in the molting rate correlated with the total weight gain (Table-5). With regard to FCR, control and treatment shrimp consumed 147.13 ± 2.09 and 151.75 ± 1.04 g of total feed, respectively. Feed consumption did not differ significantly between treatments; however, the FCR of the control shrimp (2.51 ± 0.09) was significantly higher (p < 0.05) than that of the treated shrimp (1.55 ± 0.04). This 1.62-fold difference in FCR indicates a higher feed utilization efficiency under supersaturated DO conditions.

### Effect of supersaturated DO on shrimp hemocytes and gene expression

#### Shrimp hemocytes

Six hours after injection with bacterin against *V. parahaemolyticus*, THC in the control group was 1.19 ± 0.07 × 10^6^ cell/mL, which was significantly higher than that in the treatment group (1.60 ± 0.07 × 10^6^ cell/mL; p < 0.05) (Table-6). DHCs indicated that the number of granular hemocytes under supersaturated DO was significantly higher than that in the control group (p < 0.05). However, hyaline cells were significantly lower in the treatment shrimp than in the controls (p < 0.05; Table-6).

**Table-6:** Total and differential count of hemocytes in shrimp hemolymph after formalin-deactivated bacterin injection for 6 h. Samples from eight shrimp of each group were analyzed using triplicates (shown are the means ± standard deviation).

Types of hemocytes	Control (DO 5 mg/L)	Treatment (DO 15 mg/L)
Hyaline cells (%)	86.17 ± 2.29^a^	81.50 ± 2.78^b^
Granular hemocytes (%)	13.83 ± 2.28^a^	18.50 ± 2.78^b^
Total hemocyte count (10^6^ cells/mL)	1.19 ± 0.07^a^	1.60 ± 0.07^b^

^a-b^Different superscript letters in rows indicate significant differences (*t*-test). DO=Dissolved oxygen

#### Gene expression

**Figure-2:**
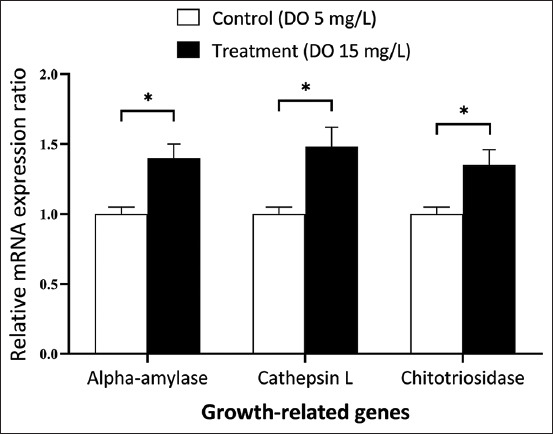
Effect of supersaturated dissolved oxygen (DO) on relative mRNA expression levels of genes related to growth in *Litopenaeus vannamei*). The amount of alpha-amylase, cathepsin L, and chitotriosidase are normalized by β-actin transcript levels. Each bar represents mean ± standard error calculated from eight individual shrimp; one asterisk (*) indicates a statistically significant difference (p < 0.05).

**Figure-3:**
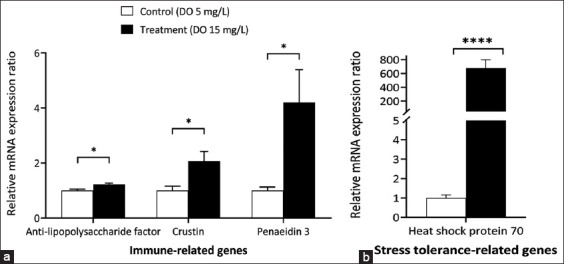
Effect of supersaturated dissolved oxygen (DO) on relative mRNA expression levels of genes related to immune and stress tolerance of *Litopenaeus vannamei*. (a) Immune-related genes and (b) stress tolerance-related gene in hemocytes of *L. vannamei* after bacterin injection. Each bar represents mean ± standard error calculated from eight individual shrimp. One asterisk (*) indicates a statistically significant difference p < 0.05; Four asterisks (****) indicate a statistically significant difference at p < 0.0001.

Alpha-amylase, cathepsin L, and chitotriosidase were considered growth-related genes. The relative ratios of these growth-related genes were significantly higher in the treatment group than in the control group (p < 0.05; Figure-2). Immune-related genes (anti-lipopolysaccharide factor [ALFs], crustin, and penaeidin3) were also significantly higher in the treatment group than in the control group (Figure-3a). Moreover, stress tolerance-related gene (Hsp70) expression was significantly higher in supersaturated DO treatments (679.04 ± 120-fold, p < 0.05; Figure-3b).

## Discussion

Optimization of DO is an essential aspect of water quality management in aquaculture ponds [40]. DO concentrations of 4–5 mg/L or higher are ideal for aquaculture production [41]. In this study, we determined the effects of supersaturated DO (15 mg/L) on the cultivation of Pacific white shrimp. This result can be attributed to the efficiency of the filtration system, which efficiently removes solid waste using synthetic wool. TSS concentrations of approximately 6.75–13.00 mg/L were observed in both groups. Typically, TSS in aquaculture ponds are primarily waterborne organic particles suspended in water. TSS decomposition shows a high oxygen demand and decreases the DO concentration in culture systems [42].

The average concentrations of ammonia, nitrite, and nitrate did not differ significantly between the treatment and control tanks (Table-4). DO plays an important role in nitrification. Elevated oxygen concentrations enhance water quality by supporting nitrifying bacteria that are crucial for the nitrogen cycle in aquatic environments. Oxygen-dependent aerobic bacterial processes require the maintenance of high DO concentrations for efficient conversion of ammonia to nitrate. Nitrate, a less toxic form of nitrogen, which can be assimilated or removed in water changes, thus improving water quality. Consequently, shrimps reared under high-oxygen concentrations exhibited improved health, lower stress, and enhanced immune responses, leading to superior growth compared to those reared under low-oxygen concentrations.

This phenomenon is likely due to the high concentration of oxygen in both treatment and control tanks, which exceeds 5 mg/L and is therefore optimal for the nitrification process when accompanied by a sufficient inorganic carbon supply through high NaHCO_3_ alkalinity [43]. Complete nitrification can reduce the levels of ammonia and nitrite, which are known to have a negative impact on growth compared to those reared at low oxygen concentrations.

Significantly higher shrimp growth in the treatment tanks illustrated the benefit of supersaturated DO for a longer cultivation time. This is consistent with the results of the previous studies by Rahmawati *et al*. [10]; however, our results demonstrated that supersaturated DO enhances shrimp molting rate by 1.55-fold, consequently improving their growth rates. Shrimp in the treatment group molted an average of seven to 8 times in 30 days, whereas shrimp in the control group molted only 5 times. Other growth parameters, such as ADG (0.22 vs. 0.13 g/day) and FCR (1.55 vs. 2.51), were better in the treatment than in the control tanks. In the present study, FCR was 1.62-fold lower in the treatment group than that in the control group. This is another advantage of enhancing shrimp yield using supersaturated DO.

In addition to beneficial effects on growth and water quality, our results suggest that supersaturated DO enhances the expression of physiological response genes. The expression of chitotriosidase was 1.35-fold higher in the treatment group than in the control group among growth-related genes. In addition, alpha-amylase and cathepsin L, which are involved in the hydrolysis of polysaccharides and proteins, respectively [44], were found to be higher in treatment shrimps. Total hemocytes play a crucial role in the innate immune system [45] and were found to be 27% more abundant in the treatment shrimp than in the control shrimp. These results indicate the potential for higher innate immune activation in shrimps. Crustacean hemocytes are broadly classified into granular hemocytes and hyaline cells based on the presence of granules in their cytoplasm [46]. The granular hemocyte count and hyaline cell abundance were approximately 33% and 5% higher in the treatment group than in the control group.

After bacterin injection, the expression of immune-related genes was correlated and consistent with an increase in THCs, especially granular hemocytes. Granular hemocytes or granulocytes contain various antimicrobial peptides such as ALFs, crustin, and penaeidin. When faced with a bacterial challenge, granulocytes become active and release peptides to target and neutralize the invading microorganisms. *L. vannamei* reared under supersaturated DO showed significantly higher levels of antimicrobial peptides, including ALF, crustin, and penaeidin 3, which are involved in bacterial pathogen defense [47–49]. Lipopolysaccharide inhibitors are released into the hemolymph during granular degeneration in response to bacterial LPS [50]. Similarly, crustin gene expression has been reported to be associated with phagocytosis and increased in hemocytes after being challenged with bacteria [51]. The penaeidin gene expression was higher in the treatment group because penaeidins are synthesized and stored in granulocytes [52].

Hsp70 is necessary for cellular processes and is considered a response gene [53]. Hsp70 expression is substantially upregulated in response to stress and bacterial or viral infection in white shrimp hemocytes and hepatopancreas [54–57]. Furthermore, Hsp70 is responsive to WSSV infection and is likely involved in antiviral defense, presumably through modulation of the pro-phenol oxidase system and apoptosis [58]. Hsp70 expression in white shrimp is temporally increased following WSSV infection that this may explain the high expression of Hsp70 after vaccination (679.04 ± 120-fold higher) in the treatment group compared to the control group. These findings support the hypothesis that supersaturated DO enhances stress tolerance and immune responses in white shrimp.

## Conclusion

Recirculating shrimp culture with oxygen microbubbles that provide 15 mg/L DO can be used for long-term shrimp cultivation. The results of our study showed that supersaturated DO enhances shrimp production in terms of growth, molting, feed conversion efficiency, and survival rate. Physiological response assay using hemocyte count and expression of immune, growth, and stress-related genes indicate that supersaturated DO benefits shrimp. These results illustrate the potential of supersaturated DO in aquaculture, particularly in RAS.

## Authors’ Contributions

CP: Conceptualization, laboratory tests, data analysis and interpretation, manuscript drafting, editing, and revision. SP: Data and sample collection, laboratory tests, data analysis and interpretation, critical review, and manuscript drafting. SD: Conceptualization, drafting, and manuscript revision. IH: Supervised the study and edited the manuscript. SW: Designed and supervised the study and drafted the manuscript. SorP: Conceptualization, supervision, and manuscript editing. All authors have read, reviewed, and approved the final version of the manuscript.
